# Operative treatment for femoral shaft nonunions, a systematic review of the literature

**DOI:** 10.1007/s11751-013-0168-5

**Published:** 2013-07-27

**Authors:** Matthijs P. Somford, Michel P. J. van den Bekerom, Peter Kloen

**Affiliations:** 1 Department of Orthopaedic Surgery, Academic Medical Centre, Meibergdreef 15, P.O. Box 22660, 1105 AZ Amsterdam, The Netherlands; 2 OLVG, Amsterdam, The Netherlands

**Keywords:** Nonunion, Pseudarthrosis, Review, Femur

## Abstract

The objective of this article is to systematically review the currently available literature to formulate evidence-based guidelines for the treatment of femoral shaft nonunions for clinical practice and to establish recommendations for future research. Articles from PubMed/MEDLINE, Cochrane Clinical Trial Register, and EMBASE, that presented data concerning treatment of nonunions of femoral shaft fractures in adult humans, were included for data extraction and analysis. The search was restricted to articles from January 1970 to March 2011 written in the English, German, or Dutch languages. Articles containing data that were thought to have been presented previously were used once. Reports on nonunion after periprosthetic fractures, review articles, expert opinions, abstracts from scientific meetings, and case reports on 5 or fewer patients were excluded. The data that were extracted from the relevant articles included: type of nonunion, type of initial and secondary treatments, follow-up, union rate, and general complications. Most studies had different inclusion criteria and outcome measures, thus prohibiting a proper meta-analysis. Therefore, only the union rate and number of complications were compared between the different treatments. Methodological quality was assessed by assigning levels of evidence as previously defined by the Centre for Evidence-Based Medicine. This systematic review provides evidence in favour of plating if a nail is the first treatment; after failed plate fixation, nailing has a 96 % union rate. After failed nailing, augmentative plating results in a 96 % union rate compared to 73 % in the exchange nailing group.

## Introduction

Since the introduction of intramedullary (IM) nails around 1939 by Küntscher, the treatment of long bone fractures has dramatically changed [1]. When Küntscher’s technique became known worldwide, 500 patients had already been treated with this method, mostly soldiers [2].

Since then, several studies have provided data which seem to favour reamed over unreamed nailing to decrease the risk of developing a nonunion in the primary treatment, but nevertheless this specific issue remains under debate [3, 4]. In the case of a nonunion, however, there is little evidence for the optimal treatment.

The objective of this article is to systematically review the currently available literature to formulate evidence-based guidelines for the treatment of femoral shaft nonunions for clinical practice and to establish recommendations for future research.

### Nonunion definition

The US Food and Drugs Administration (FDA) defines a nonunion as a fractured bone that has not completely healed within 9 months of injury and that has not shown progression towards healing over 3 consecutive months on serial radiographs [5]. The exact time frame likely differs per fractured bone and location within the bone, soft tissue condition, and fracture type.

Radiographically, a nonunion is defined by the presence of the following criteria: absence of bone trabeculae crossing the fracture site, sclerotic fracture edges, persistent fracture lines, and lack of progressive change towards union on serial radiographs. The presence or absence of callus is not a criterium since this depends on the site of the fracture, and whether there is primary or secondary bone healing involved. Furthermore, there should be persistent pain, or even motion at the fracture site. This is best elicited by weight bearing.

The objective of this article is to systematically review the currently available literature to formulate evidence-based guidelines for treatment of femoral shaft nonunions for clinical practice and recommendations for future research.

## Materials and methods

### Inclusion and exclusion criteria

All titles and abstracts of relevant studies were reviewed with a set of predefined inclusion and exclusion criteria. All articles from January 1970 onward that presented data concerning treatment of nonunions of femoral shaft fractures were included for further data extraction. In general, a delayed union is defined as no fracture healing after 6 months and nonunion is defined a no fracture healing after 9 months with no radiological progression for 3 consecutive months. The definition of a nonunion or delayed union differed per article, and sometimes no time until diagnosis of a nonunion was provided. All primary and delayed/nonunion treatments were included. Septic and aseptic nonunions were included. The diagnosis of delayed or nonunion was made with history, physical examination, and radiographs or CT-scanning. Studies concerning several types of nonunions were included if the femoral shaft nonunions could be evaluated separately.

Reports on nonunion after periprosthetic fractures were excluded. Review articles and expert opinions were excluded because these articles do not report on new patient series. Abstracts from scientific meetings that were not published as a full-text article were also excluded, as were case reports on 5 or less patients. The search was restricted to articles written in the English, German, and Dutch languages. Articles presenting data that were thought to have been presented previously were used once.

### Identification of studies

A comprehensive literature search was performed with the assistance of a clinical librarian, using the following Mesh search terms: femur, nonunion, delayed union, pseudarthrosis, fracture, trauma, injury, healing, treatment, and complication (Table 1). The search was limited to adult humans in the following databases: PubMed/MEDLINE, Cochrane Clinical Trial Register, and EMBASE. Studies were searched in the period from January 1970 to March 2011. The obtained reference list of retrieved publications was manually checked for additional references potentially meeting the inclusion criteria and not found by the electronic search.

**Table 1:** Search query used in this systematic review, including the limits

((“Femoral Fractures”[Mesh]) OR (femur AND fracture*) OR (femoral AND fracture*)) AND (midshaft OR shaft OR diaphyseal) AND (ununited OR union delay OR Fracture Healing OR pseudarthrosis OR delayed union* OR delayed union OR nonunion* OR nonunion* OR nonunion*) Limits: Humans, English, German, Dutch, All adult: 19 + years	

From the title abstract, two reviewers (MS and MB) independently reviewed the literature searches to identify relevant articles for full review. From the full text, using the above-mentioned criteria, the reviewers independently selected articles for inclusion in this review. Disagreement was resolved by group discussion, with arbitration by the senior author (PK) where differences remained. Studies were not blinded for author, affiliation, and source. Excluded articles are listed in Table 2.

**Table 2:** Excluded articles with their exclusion reason

Years	Author	Reason for exclusion
1969	Werner	Case report
1972	Esah	Case report
1975	Kostuik	Comparison of several treatments
1984	Müller	Analysis bridgeplate, no patient information
1985	Slatis	5 cases
1986	Johnson	Double serie
1986	Kreusch	Femur and tibia, mixed group
1986	Klemm	Primary treatment
1990	Wood	5 cases
1990	Blatter	Case report
1992	Johnson	Comparison of several treatments
1992	Hou	5 cases
1997	Wei	No nonunion
1998	Ueng	5 cases
1998	Ueng	5 cases
1998	Johnson	Double serie
2000	Giannoudis	No intervention
2000	Kim	<5 patients with femur nonunion
2001	Devnani	Location not mentioned
2001	Bellabarba	Double serie
2002	Ebraheim	Case report
2002	Pihlajamäki	Comparison of several treatments
2002	Menon	<5 patients with femur nonunion
2003	Brinker	5 cases
2003	Canadian Orthopaedic Trauma Society	No nonunion
2003	Wu	Associated femoral neck fracture
2007	Crowley	Review
2007	Alt	1 case and double fracture
2007	Morasiewicz	Femur and tibia, mixed group
2009	Prasarn	5 cases
2009	Taitsman	No intervention
2010	Wedemeyer	Case report
2011	Wedemeyer	Case report
2011	Kim	Classification

### Data extraction

After the initial assessment for inclusion, the following data were extracted from the included articles selected: (a) septic nonunion, type of initial and secondary treatments, follow-up, union rate, and general complications.

After initial data extraction, the exclusion criteria were reassessed. It became clear that most studies had different inclusion criteria and outcome measures, thus prohibiting a proper meta-analysis and comparison between the different studies. Only the union rate and number of complications were compared between the different treatments.

### Methodological quality

Methodological quality of included studies was assessed by assigning levels of evidence as previously defined by the Centre for Evidence-Based Medicine (http://www.cebm.net). In short, for studies on therapy or prognosis, level I is attributed to well-designed and performed randomized controlled trials, level II to cohort studies, level III to case control studies, level IV to case series, and level V to expert opinion articles (Table 3). Levels of evidence were assigned by two authors (MS and MB). Disagreement was resolved by group discussion. Based on the levels of evidence, some recommendations for clinical practice were formulated. A grade was added, based on the evidence supporting that recommendation. Grade A meant treatment options were supported by strong evidence (consistent with level I or II studies); grade B meant treatment options were supported by fair evidence (consistent with level III or IV studies); grade C meant treatment options were supported by either conflicting or poor quality evidence (level IV studies); and grade D was used when insufficient evidence existed to make a recommendation (Table 4).

**Table 3:** Level of evidence

Level I: High-quality prospective randomized clinical trial Level II: Prospective comparative study Level III: Retrospective case control study Level IV: Case series Level V: Expert opinion

**Table 4:** Grades of recommendation given to various treatment options based on the level of evidence

Evidence supporting that treatment Grade A: Treatment options are supported by strong evidence (consistent with level I or II studies) Grade B: Treatment options are supported by fair evidence (consistent with level III or IV studies) Grade C: Treatment options are supported by either conflicting or poor quality evidence (level IV studies) Grade D: Insufficient evidence exists to make a recommendation	

## Results

**Fig. 1:**
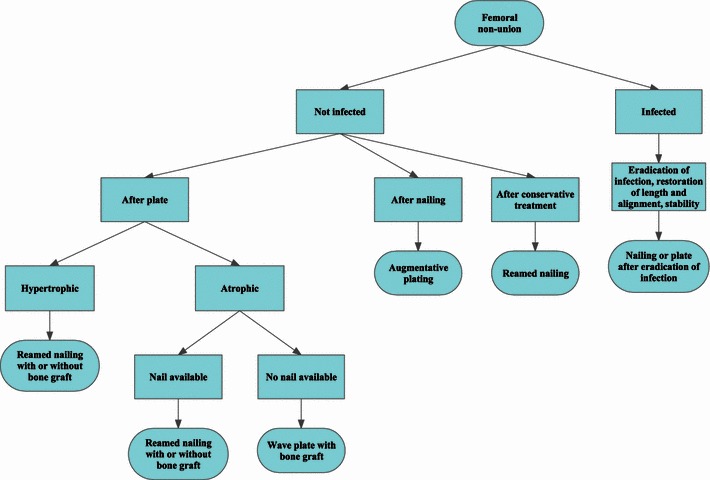
Proposed decision chart for the treatment of femoral non-union

Through database search, 71 articles were eligible for analysis. By manual reference checking, an additional 24 articles were included. After removal of 3 duplicates, 92 abstracts were screened. Ten articles were excluded based on the aforementioned criteria. The full text of the remaining 82 articles was assessed. This resulted in an additional 25 articles being excluded because of the aforementioned criteria. Eventually, 57 articles were included in our analysis (Fig. 1).

The results of exchange nailing were described in 11 [6, 7] patient series concerning 343 patients with a union in 251 patients (73 %) and an average union time of 7 months. Six complications were described.

The results of augmentative plating were described in 5 studies concerning 121 patients with a union in 118 patients (98 %) and an average union time of 6 months. One complication was described.

The results of nailing after initial plating were described in 5 patient series concerning 99 patients with a union in 95 patients (96 %) and an average union time of 6 months. Fourteen complications were described.

Thirty-four articles describe a technique that could not be classified in one the previous treatment categories (Fig. 1).

## Discussion

Based on the systematic review of the currently available and relevant literature, we can formulate evidence-based guidelines for treatment of femoral shaft nonunions for clinical practice, as well as some recommendations for future research.

### Dynamization

Dynamization is the removal of those interlocking screws that have initially statically locked an IM nail. This technique has been proven beneficial for example in tibial fracture healing [8]. However, the data remain conflicting with respect to the potential role of dynamization in femoral fracture healing [9, 10]. To the best of our knowledge, no (randomized) comparative trial of dynamization alone versus other techniques has been performed. Auto-dynamization, the breakage of the screws of a statically locked nail, has been described, but concerns only a subgroup of nonunions. Complications of dynamization include shortening of the affected limb.

### Recommendation grade D

#### Reamed nailing after plate

Placing an IM nail after primary non-operative treatment was initially only used for midshaft femoral nonunions. The introduction of locking nails allowed reamed nailing to also be used for non-isthmal femoral nonunions.

A total of 99 patients from our systematic review were treated with a nail after primary plating distributed over 4 studies [11–14]. Average healing time was 6 months with a healing rate of 96 % (n = 95). Complications described were limited to nonunion after the secondary surgery. Emara et al. [13] did not find a difference in outcome if an additional autologous bone graft was used in a randomized trial (Table 5).

**Table 5:** Nailing after plate. Nonunions are not separately listed as complications

Years	Author	Number of patients	Primary treatment	Secondary treatment	Complications	Union rate n (%)
1999	Wu	21	Plate	Reamed nail	Not mentioned	21 (100)
2001	Wu	8	Plate	Nailing + bone graft	0	7 (93)
2008	Emara*	20	Plate	Nailing + bone graft	5	20 (100)
2008	Emara*	20	Plate	Nailing	1	20 (100)
2009	Megas	30	Plate	Nailing	8	27 (91)

Time to union (months)	Remarks
6	
4 (3–6)	
4.8 ± 1.15	RCT
4.9 ± 1.33	RCT
7.9 ± 3.3	

* One study, divided in two groups to show the results of grafting or no grafting

### Recommendation grade C

#### Exchange reamed nailing

If initial treatment with an IM nail results in a nonunion, the nail can be removed and a larger diameter nail can be placed after overreaming. The presumed causes of healing after exchange nailing are both biological and mechanical [5]. The biological effects believed to be that reaming increases periosteal blood flow, whereas it decreases endosteal vascularization. The periosteum reacts to increased blood flow with new bone formation. Products of the reaming itself contain osteoblasts and possibly multipotent stem cells as well as growth factors that play a role in bone healing.

The mechanical effects of reaming are that a larger diameter nail (preferably >2 mm thicker) provides greater bending rigidity and strength than the original nail. Reaming also increases the length of the isthmus providing a better endosteal purchase of the new nail. Increased stability can also be obtained by placing a longer nail than before and by using a nail that allows for more interlocking holes and/or holes that are not parallel. Most recent advances are the option for locking nail implants that might provide increased stability.

In hypertrophic nonunions treated with exchange nailing, the increased stability will be sufficient for healing. For atrophic nonunions, it is thought that the reaming debris will augment bone healing. For nonunions treated with exchange nailing, there is a possible additional benefit from open bone grafting which might result in shorter union times [15].

Our systematic review resulted in 343 patients treated with exchange nailing in 11 studies [6, 7, 15–23]. Union was seen in 73 % (n = 251) at an average of 7 months. Of the complications reported, there were 2 failed nails and 2 infections. Of note is that recent studies have a lower success rate after reamed exchange nailing after one procedure than previous reports. We believe this is caused by the more liberal indications for reamed nailing and the type of nonunion (hypertrophic vs. atrophic) [24] (Table 6).

**Table 6:** Reports on exchange nailing. Nonunions are not separately listed as complications

Years	Author	Number of patients	Primary treatment	Secondary treatment	Complications	Union n (%)
1997	Wu	35	IM nail	Exchange nailing	0	35 (100)
1999	Wu^*^	8	IM nail	Exchange nailing	0	8 (100)
1999	Wu*	15	IM nail	Nailing and bone graft	0	15 (100)
1999	Furlong	25	IM nail	Exchange nailing	0	24 (96)
2000	Weresh	19	IM nail	Exchange nailing	0	10 (53)
2000	Hak	23	IM nail	Exchange nailing	Not mentioned	18 (78)
2002	Wu	36	IM nail	Exchange nailing	0	33 (92)
2002	Yu	36	IM nail	Exchange nailing	0	36 (100)
2003	Banaszkiewicz	19	IM nail	Exchange nailing	2 infection, 2 failed nails, 2 delayed union	11 (58)
2005	Wu	11	IM nail	Exchange nailing	0	9 (80)
2007	Wu^#^	34	IM nail	1 mm overreaming	0	31 (91)
2007	Wu^#^	40	IM nail	>2 mm overreaming	0	37 (93)
2009	Shroeder	42	IM nail	Exchange nailing	0	36 (86)

Time to union (months)	Remarks
4	Pseudo-RCT
4.4 ± 0.9	
5.7 ± 1.5	
7	
?	
–	All 5 nonunions in smokers
4 (3–8)	
4 (3–8)	
9	
4	Broken screws and shortened >1.5 cm, one death because of other reason
4 (3–6)	
4 (3–8)	
4	

* One study, divided in two groups to show the results of grafting or no grafting

^#^One study, divided in two groups to show the results of difference in the amount of overreaming

Wu et al. [22] published a retrospective comparison of reaming 1 or >2 mm greater than the previous nail. This resulted in comparable union rates after a comparable time.

There is no consensus whether open bone grafting is beneficial in reamed exchange nailing for a nonunion. If residual instability is present, a locked augmentation plate can be placed [24, 25].

### Recommendation grade C

#### Augmentative plate fixation

Failure of exchange reamed nailing has been noted in nonunions with extensive comminution, large segmental defects, and metaphyseal–diaphyseal nonunions [18, 21]. Leaving the intramedullary nail in situ when plating a nonunion, i.e. augmentative plating, has been reported for humeral, tibial, and femoral nonunions [26]. This approach uses the load-sharing capacity of the nail with good axial and bending strength, while the plate provides additional rotational control. A retrospective study by Park et al. [27] showed, be it in small groups, that augmentative plating gave better outcomes than exchange nailing for non-isthmal femoral nonunions.

From our systemic review, we found 122 patients in 5 studies treated with augmentative plating [26, 28–31] 96 % (n = 118) healed in an average of 6 months. No complications were reported (Table 7).

**Table 7:** Reports on augmentative plating. Nonunions are not separately listed as complications

Years	Author	Number of patients	Primary treatment	Secondary treatment	Complications	Union rate n (%)
1997	Ueng	17	IM nail	Augmentative plate	Not mentioned	17 (100)
2005	Choi	15	IM nail	Augmentative plating + bone graft	0	15 (100)
2008	Nadkarni	7	IM nail	Augmentative plate	0	7 (100)
2008	Roetman	32	IM nail	Augmentative plate	0	29 (91)
2010	Chen	50	IM nail	Augmentative plating + bone graft	1	50 (100)

Time to union (months)	Remarks
7 (6–10)	
7 (5–11)	
7 (6–8)	
5	
6 (4.5–8)	8 distal and 7 proximal fractures

Prior to the availability of locking plates (that can rely on unicortical fixation), this technique was quite challenging given the need for bicortical screw purchase. However, locking plates have substantially facilitated augmentative plating from a surgical technique perspective.

Removing the locking screws in the nail will even allow compression with the AO tensioner device prior to augmentative plating. Finally, the use of additional bone grafting in augmentative plate fixation is variable [16, 28–31].

An obvious shortcoming of this technique is that it does not allow for correction of deformity with the presence of an intact nail.

### Recommendation grade C

#### Plate fixation

Before the introduction of reamed exchange nailing, the use of compression plating for femoral shaft nonunions was the gold standard. The plate functions as a tension band on the lateral side. As such, it will also help with correction of malalignment. The bone itself absorbs the axial compressive forces. In their book on nonunions, Weber and Čech [25] advocate debridement, sequestrectomy, use of plates for “mechanical rest” and “massive cancellous autograft”.

In the recent AO book on nonunions, these are listed as still valid principles [32]. When there is a medial bony defect, a standard plate is subjected to a local concentration of bending forces which may induce failure. For these specific nonunions, the wave plate was introduced by Blatter and Weber [33]. The plate has a contour in its midportion so that it stands away from the bone at the abnormal area. The wave is believed to preserve local blood supply to the bone at the site of the nonunion and provides more space for grafting. The wave can share axial loads more effectively. Combined with the indirect reduction techniques using an AO femoral distractor, this technique can be considered “biological”. In two large retrospective series of femoral shaft nonunions, the wave plate led to union after a single surgery in the vast majority of cases [e.g. 41 of 42 cases (98 % union rate) [34] and 64 of 75 cases (85 % union rate) [35]]. Schulz et al. also included nonunions after osteotomies. The complications reported were 2 infections and 9 nonunions.

### Recommendation grade C

#### Remaining papers

Only scarce literature exists on the treatment of infected femoral shaft nonunions. In general, the treatment goals for these nonunions are: eradication of infection, restoration of length and alignment, bone healing, and optimal functional outcome [36].

There remained a considerable amount of other treatments, obsolete treatments, or reports which were too heterogeneous to draw conclusions from [24, 37–61] (Table 8).

**Table 8:** Remaining included studies

Years	Author	Number of patients	Primary treatment	Secondary treatment
1975	Oh	15	Several	Nailing
1984	Harper	16	Several	Fluted rod
1985	Heiple	25	16 conservative, 7 plate, 1 IM nail	Fluted rod
1986	Webb	101	Several	Reamed nail
1986	Kempf	27	Several	Reamed nail
1987	Jupiter	7	1–9 earlier operations	Vascularized fibula graft
1988	Johnson	6	Several	Exchange nailing
1988	Barquet	13	Several	AO tubular external fixation
1992	Wu	64	Several	Exchange nailing
1992	Wu	20	Several	Exchange nailing
1992	Wu	17	Several	Nailing and lengthening
1996	Meng-Hai	16	11 plate, 5 IM nail	Cast with BMP in the bone
1996	Weise	28	Several	Reamed nail
1996	Matelic	7	Several	Lateral and endosteal plating
1997	Ring	42	21 patients more than 1 operation	Wave plate
1997	Cove	44	Several	43 plaat, 1 nagel, 8 ook fibula
1999	Bungaro	7	IM nail	Plate + graft, removal of nail
1999	Wu	56	Several	Locked nail, graft and lengthening
2000	Richter	145	112 plate, 24 IM nail, 9 external fixation	Several
2000	Johnson	30	Several	Cortical bone carrier with BMP
2001	Bellabarba	23	IM nail	Angled blade plate
2001	Rompe	24	Several	Extracorporeal shockwave
2002	Finkemeier	39	Several	Reamed nail
2004	Abdel-Aa	16	Plate	Plate
2004	Wu	32	Several	Nailing and grafting
2005	Inan	11	IM nail	Cyclic compression and distraction
2007	Niedzwiedzki	22	Several	Nailing (+bone graft in 9 patients)
2008	Oh	32	2 plate, 12 IM nail, 1 conservatively	Exchange nailing
2009	Steinberg	16	Several	Expandable nail
2009	Schulz	75	3 conservative, 5 external fixation, others plate	Wave plate
2010	Blum	50	Several	Distraction osteogenesis (+bone graft in 15 patients)
2010	Park	18	IM nail	7 exchange nailing, 11 augmentative plating
2010	Benazzo	7	Several	(Exchange) nailing

Complications	Union rate n (%)	Time to union (months)	Remarks
0	15 (100)	–	
1 infection	12 (75)	7 (2–12)	
0	24 (96)	–	
2 migration of nail, 2 pain, 2 rotation deformity	101 (96)	5	
2 infection	25 (93)	4	
0	5 (71)	4–6	
0	6 (100)	5	
4	–	9	Infected nonunions
1 infection, 3 failed nails	58 (91)	3–5	
1 infection, 1 plate failure	12 (60)	4	
2	17 (100)	5	
0	15 (94)	6	
0	26 (93)	–	
3	6 (88)	19.2 (15–36)	1 pertrochanteric femur fracture
0	35 (100)	5,1	Pseudo-RCT
2 infection, of which 1 amputation	41 (98)	–	
1 amputation, 17 > 2 cm shortening, 10 loss of knee flexion	33 (75)	12	
0	7 (100)	4 (3–7)	
0	44 (78)	4 (3–8)	
–	79 (55)	–	
6 plate failure	24 (80)	6 (3–9)	
2 plate failure	21 (91)	4 (2.5–6)	
0	18 (75)	4 (2–7)	Osteotomy and fracture
1 TE, 1 infection	29 (74)	19 (4–75)	
2	15 (91)	4.9 (4–6)	3 infected nonunions
0	31 (97)	4.5 (3–6)	1 hip disarticulation
1 osteomyelitis, 11 pain, 12 pintract infection, 1 bacterial arthritis, 1 broken pin	11 (100)	6 (5–8)	
–	20 (92)	–	
1 dynamization	31 (97)	–	1 infected nonunion
1 infection	14 (88)	4	
3 plate failures	64 (85)	7	Osteotomy and fracture
63	50 (100)	20.7 (12–35)	Infected nonunion w bone defect
0	EN 2(28), AP 11(100)	7 (4–16)	
1 heterotopic ossification	6 (86)	7	

*TE* thromboembolic event

## Conclusions

Care should be taken in interpreting these results since the overall grade of recommendation did not exceed grade C, meaning weak support of the drawn conclusions. However, based on the best available evidence, we conclude that augmentative plating is the treatment of choice if an intramedullary nail is in situ (augmentative plating results in a 96 % union rate compared to 73 % in the exchange nailing group). The concept is that nonunion after nailing is in a great part of cases because of instability (hypertrophic nonunion) which is treated with providing stability. This is easier to achieve with an augmentative plate than with exchange nailing.

In case of a failed plate fixation, reamed nailing results in 96 % union rate, thus being the treatment of choice. If a plate is the only available treatment option, a wave plate should be placed to preserve blood supply at the nonunion site and to share the axial load as good as possible.

**Fig. 2:**
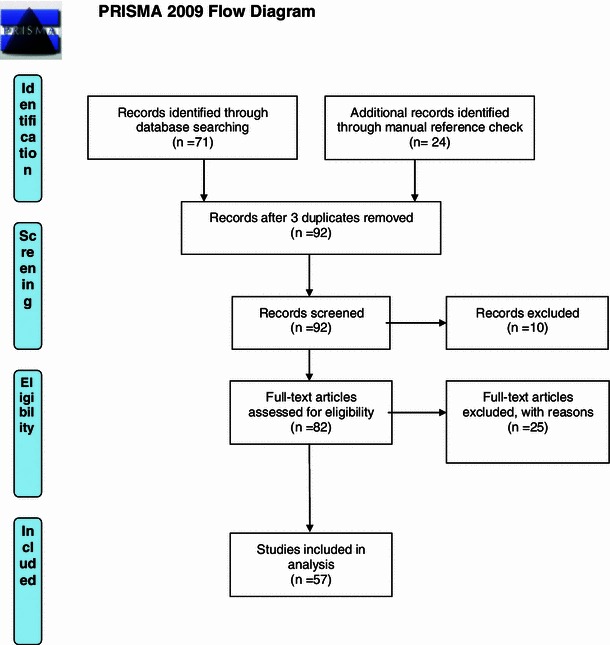
PRISMA 2009 flow diagram. *Source* Moher et al. [62]

With the recommendations from our review, we propose a decision diagram for treating femoral nonunions. Where no evidence is present we included our own experiences (Fig. 2).
